# Chemoprevention of Rat Mammary Carcinogenesis by Apiaceae Spices

**DOI:** 10.3390/ijms18020425

**Published:** 2017-02-16

**Authors:** Farrukh Aqil, Jeyaprakash Jeyabalan, Radha Munagala, Srivani Ravoori, Manicka V. Vadhanam, David J. Schultz, Ramesh C. Gupta

**Affiliations:** 1 Department of Medicine, University of Louisville, Louisville, KY 40202, USA; f0aqil01@louisville.edu (F.A.); r0muna01@louisville.edu (R.M.); 2 James Graham Brown Cancer Center, University of Louisville, Louisville, KY 40202, USA; j0jeya01@louisville.edu (J.J.); srivani.ravoori@aacr.org (S.R.); mvvadh01@louisville.edu (M.V.V.); 3 Department of Biology, University of Louisville, Louisville, KY 40202, USA; djschu02@louisville.edu; 4 Department of Pharmacology and Toxicology, University of Louisville, Louisville, KY 40202, USA

**Keywords:** Apiaceae spices, breast cancer, estradiol, ACI (August-Copenhagen Irish) rats, chemoprevention

## Abstract

Scientific evidence suggests that many herbs and spices have medicinal properties that alleviate symptoms or prevent disease. In this study, we examined the chemopreventive effects of the Apiaceae spices, anise, caraway, and celery seeds against 17β-estrogen (E2)-mediated mammary tumorigenesis in an ACI (August-Copenhagen Irish) rat model. Female ACI rats were given either control diet (AIN 93M) or diet supplemented with 7.5% (*w*/*w*) of anise, caraway, or celery seed powder. Two weeks later, one half of the animals in each group received subcutaneous silastic implants of E2. Diet intake and body weight were recorded weekly, and animals were euthanized after 3 and 12 weeks. E2-treatment showed significantly (2.1- and 3.4-fold) enhanced growth of pituitary gland at 3 and 12 weeks, respectively. All test spices significantly offset the pituitary growth by 12 weeks, except celery which was effective as early as three weeks. Immunohistochemical analysis for proliferative cell nuclear antigen (PCNA) in mammary tissues showed significant reduction in E2-mediated mammary cell proliferation. Test spices reduced the circulating levels of both E2 and prolactin at three weeks. This protection was more pronounced at 12 weeks, with celery eliciting the highest effect. RT-PCR and western blot analysis were performed to determine the potential molecular targets of the spices. Anise and caraway diets significantly offset estrogen-mediated overexpression of both cyclin D1 and estrogen receptor α (ERα). The effect of anise was modest. Likewise, expression of CYP1B1 and CYP1A1 was inhibited by all test spices. Based on short-term molecular markers, caraway was selected over other spices based on its enhanced effect on estrogen-associated pathway. Therefore, a tumor-end point study in ACI rats was conducted with dietary caraway. Tumor palpation from 12 weeks onwards revealed tumor latency of 29 days in caraway-treated animals compared with first tumor appearance at 92 days in control group. At the end of the study (25 weeks), the tumor incidence was 96% in the control group compared with only 70% in the caraway group. A significant reduction in tumor volume (661 ± 123 vs. 313 ± 81 mm^3^) and tumor multiplicity (4.2 ± 0.4 vs. 2.5 ± 0.5 tumors/animal) was also observed in the caraway group compared with the control group. Together, our data show dietary caraway can significantly delay and prevent the hormonal mammary tumorigenesis by modulating different cellular and molecular targets.

## 1. Introduction

An estimated 252,710 new cases of invasive breast cancer will be diagnosed in 2017 [1]. As such, breast cancer (BC) is second only to skin cancer as the most common cancer among women in the United States and represents approximately 29% of newly diagnosed cancers in women [1]. Despite new and more effective treatments coupled with improvements in early detection, 40,890 people (40,450 women and 440 men) are estimated to have died from BC in 2016 [1]. Major contributing factors to BC include reproductive factors, environmental exposures, diet and life style, and hormone replacement therapy (HRT), and thus a significant population of women are still at risk for developing BC [2,3]. Recent research findings suggest breast density is an important predictor of a woman’s BC risk. Women with high breast density are four to five times more likely to get BC than women with low breast density [4,5,6]. New molecular breast imaging (MBI) technology [7] can readily detect small tumors or suspected tumors in dense breast tissue and add more women to the “watch and wait” list, while increased genetic testing can identify women with BRCA1 and BRCA2 mutations that elevate risk for developing BC [8]. Prophylactic mastectomy can reduce the risk of developing BC by nearly 90% for women with these genetic mutations [9,10,11]. However, it is not known whether this risk reduction translates into longer survival for the patient [11]. Moreover, this is a drastic and irreversible choice. Because breast cancer is a major health problem for all women, effective prophylactic prevention strategies are an attractive addition to the established roles of early screening, detection, and treatment.

**Figure 1:**
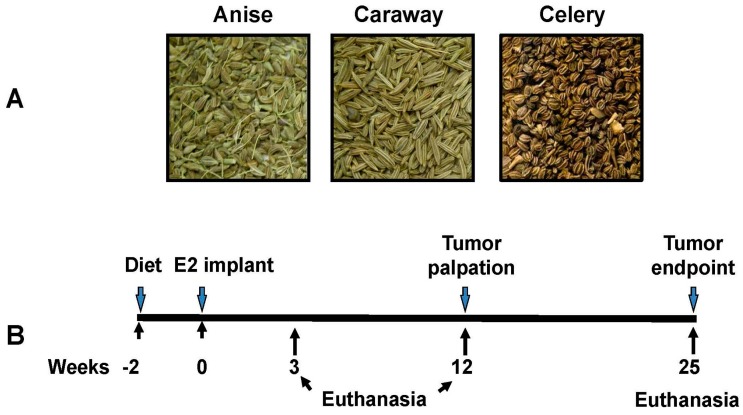
Apiaceae spice seed samples (Anise, Caraway, and Celery) used in this study (**A**); and schematic representation of animal study plan (**B**).

Interestingly, more than 50% of BC patients and high-risk women are estimated to use some form of alternative medicine [12,13]. Clinically, preventive measures often target estrogen (17-β-estradiol or E2) or E2 metabolism. Selective E2-receptor modulators (SERMs), such as tamoxifen and raloxifene, are approved for both treatment and prevention of BC and are reported to reduce BC risk by approximately 35% [14]. However, clinicians rarely use tamoxifen for prevention due to serious side effects such as uterine cancer [15,16]. Raloxifene is less likely to cause uterine cancer but has other adverse effects, such as cataracts and increased risk of blood clots [14]. A large number of women are also exploring phytoestrogens that may function as “natural SERMs” [17] as well as other natural products such as indole-3-carbinol and tea catechins [18]. However, tea catechins and many other plant bioactives suffer from poor oral bioavailability. There is clearly a demand and “unmet need” for new plant bioactives that are efficacious to prevent BC in high-risk women, achieve high oral bioavailability, and are safe for prolonged periods. Commonly used spices provide a rich array of phytochemicals that may reduce the risk of certain cancers [19,20]. Scientific evidence is accumulating that indicates herbs and spices have medicinal properties that alleviate symptoms or prevent disease. Caraway (*Carum carvi*), anise (*Pimpinella anisum*), and celery (*Apium graveolens*) belong to the Apiaceae family (Figure 1A), contain a high content of polyphenolics, and are demonstrated to have pharmacological activities [20,21,22]. These spice seeds have extensive dietary exposure, as they are major ingredients of curry powders and many savory spice mixtures used in Indian cuisine and for flavoring curries, soups, sausages, cakes, etc. Despite known pharmacological properties, very little work has been carried out on the chemopreventive effects of these spices.

Historically, herbs and spices have enjoyed a rich tradition of use for their flavor-enhancement characteristics and medicinal properties. Natural plant products have been used throughout human history and have also been exploited from historic times in the treatment of cancer [23,24]. In vitro studies indicate bioactive components of herbs and spices inhibit pathways that regulate cell division, cell proliferation, and detoxification as well as inflammatory and immune response [20].

The process of tumorigenesis requires cellular transformation, hyper-proliferation, invasion, angiogenesis, and metastasis. The mechanism by which E2 induces mammary tumorigenesis is complex. E2 may exert its carcinogenic actions by at least two principal pathways: first, through stimulating cell proliferation, and second via the genotoxicity of hydroxylated metabolites of E2, particularly 4-hydroxy-17β-estradiol (4E2) or 4-hydroxyestrone (4E1), which induce mutations leading to carcinogenesis [25]. The first pathway involves modulation of cell cycle proteins by interaction with estrogen receptors (ERs) or possibly via non-genomic mechanisms [26,27]. Low levels of circulating E2 (60–120 pg/mL) are sufficient to induce mammary tumors in ACI (August-Copenhagen Irish) rats, and these tumors have been shown to resemble human ductal breast cancer histopathologically [28], suggesting an important role of E2 in mammary tumorigenesis. Thus, agents that can suppress these pathways have the potential to suppress carcinogenesis. Accumulating scientific evidence indicates that many cancers are preventable since diet and nutrition are key factors in modulating cancer risk [29]. In fact, dietary habits are estimated to contribute to at least 35% and perhaps up to as many as 70% of all human cancer cases [30,31].

Recently, we demonstrated extracts derived from the Apiaceae family are protective against oxidative DNA adducts resulting from redox activity of 4E2. We also showed inhibition of cytochrome P450s could be associated with metabolism of E2 [32]. In this current study, we expand on our previous work by demonstrating the chemopreventive potential of the Apiaceae spices, anise, caraway, and celery, against E2-induced mammary carcinogenesis using an ACI rat model.

## 2. Results

### 2.1. Body/Organ Weights and Diet Consumption

Test diets including Apiaceae spices (7.5%, *w*/*w*) did not affect food consumption or body weight gain compared to untreated animals, and thus appear to be well tolerated (Table 1). As expected following E2 treatment, the body weight of animals increased significantly compared to age-matched untreated control animals. However, at the end of the study (25 weeks), there was no significant difference between the body weight gains of the control and E2-treated animals irrespective of dietary intervention.

No significant spice intervention differences were observed in the liver, pituitary, and mammary tissue weights after, 3, 12, and 25 weeks. Liver and mammary tissue weights correlated with the body weights of the animals. After three weeks, liver weight increased significantly in the E2-treated controls (7.0 ± 0.4 g) compared to untreated control animals (4.5 ± 0.4 g). However, no difference was observed between E2-treated groups provided control or spice diets (Table 1). A similar trend was observed after 12 weeks. After three weeks, a slight but statistically insignificant increase was observed in the mammary tissue weight in the E2-treated animals. This increase was more pronounced and significant (*p* < 0.05) after 12 weeks irrespective of the dietary interventions. Spice intervention alone did not affect the pituitary gland at 3 or 12 weeks. The pituitary gland organ wet weight in the E2-treated rats was significantly increased after 3 (2-fold) and 12 (4-fold) weeks of the E2 treatment. The E2-associated increase in the pituitary gland was substantially reduced when animals were provided spice diet (Table 1).

### 2.2. Proliferation Index

**Figure 2:**
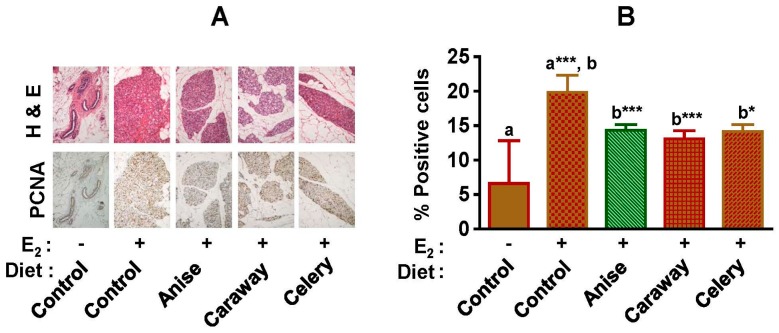
Effect of dietary Apiaceae spice intervention on the mammary cell proliferation in the absence and presence of 17β-estradiol (E2) treatment. (**A**) Representative photomicrographs (20× magnification) of normal and hyperplastic mammary tissue following immuno-histochemical staining for proliferating cell nuclear antigen (PCNA); (**B**) Percentage (average ± SD) of deeply-stained cells for PCNA in mammary tissues (*n* = 5). Symbols (– and +) denotes the absence and presence of E2, respectively. Alphabets (a and b) followed by asterisk shows statistical differences at * *p* < 0.05, *** *p* < 0.001.

The antiproliferative activity of test spices was determined by immunohistological analysis, staining the mammary tissue sections for proliferative cell nuclear antigen (PCNA) (Figure 2A). The blinded slides were read by two independent pathologists and the average of deep-stained cells was determined (Figure 2B). The E2 treatment increased the proliferation of mammary tissues by five to six-folds, while all test groups receiving spice diets significantly inhibited E2-associated mammary cell proliferation. The effect was most pronounced with the caraway intervention.

### 2.3. Effect of Spice Intervention on Serum Estrogen Levels

**Figure 3:**
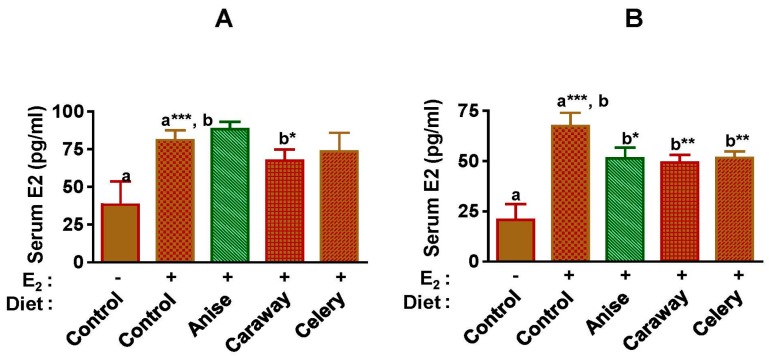
Circulating E2 levels measured at different time points in the blood plasma of the animals provided control AIN-93M diet or diet supplemented with spices (7.5% *w*/*w*) euthanized after 3 (**A**) and 12 (**B**) weeks. Data represent mean ± SD (*n* = 6). Symbols (– and +) denotes the absence and presence of E2, respectively. Alphabets (a and b) followed by asterisk shows statistical differences at * *p* < 0.05, ** *p* < 0.01, *** *p* < 0.001.

The basal level of circulating E2 was approximately 25 pg/mL in the control rats. A significant increase of nearly three-fold was observed in rats receiving E2 implants (Figure 3). Anise and celery did not affect circulatory levels of E2 at three weeks of intervention but significantly reduced the levels at 12 weeks. On the other hand, the caraway diet significantly inhibited circulatory levels of E2 at three weeks with further reduction noted at 12 weeks.

### 2.4. Circulatory Prolactin

**Figure 4:**
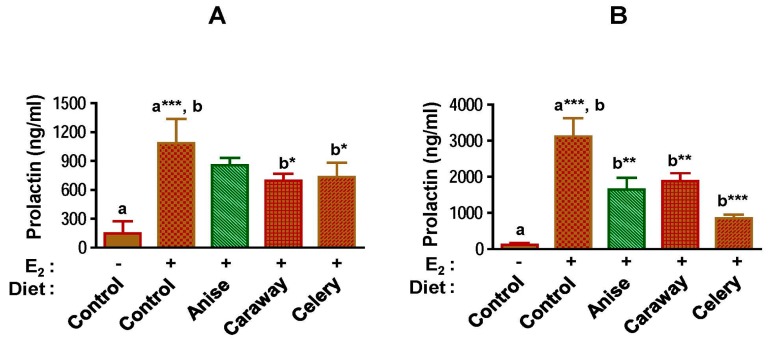
Spice diets reduce circulating prolactin levels in E2-treated ACI (August-Copenhagen Irish) rats. Prolactin levels were measured at 3 (**A**) and at 12 (**B**) weeks by enzyme immune-assay kit (Alpco Diagnostics, Windham, NH, USA) as per the manufacturer’s instructions. Average ± SD of five to six animals are shown. Symbols (– and +) denotes the absence and presence of E2, respectively. Alphabets (a and b) followed by asterisk shows statistical differences at * *p* < 0.05, ** *p* < 0.01, *** *p* < 0.001.

Increased pituitary gland weight is directly correlated with pituitary DNA content and the level of prolactin in systemic circulation [33]. Therefore, we analyzed plasma prolactin levels. No differences were found in plasma prolactin levels between control animals and animals receiving spice intervention. However, a significant increase in the prolactin levels was observed at both time-points with E2 treatment (*p* < 0.001). Prolactin levels were increased by eight- to ten-fold at 3 weeks and by almost thirty-fold at 12 weeks. This increase was significantly off-set by both caraway and celery at 3 weeks and by all test spices at 12 weeks (Figure 4). The reduction in prolactin levels was strongest with the celery diet at the 12-week point.

### 2.5. Modulation of Estrogen-Related Markers

**Figure 5:**
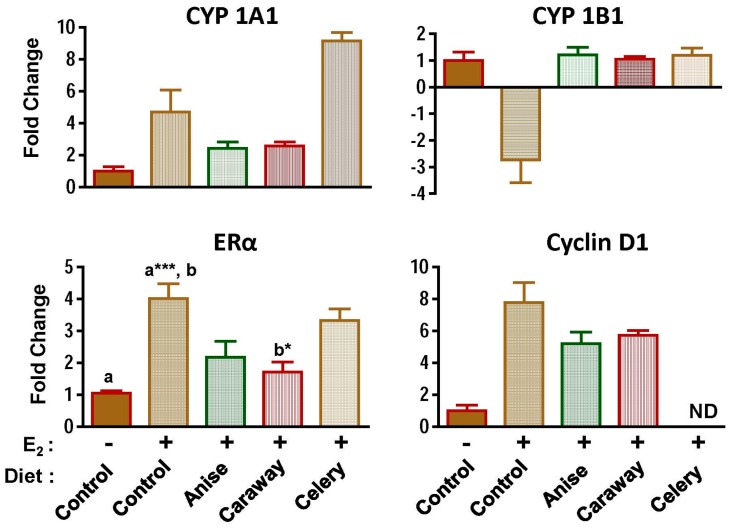
Effect of diet supplemented with anise, caraway, and celery powder (7.5% *w*/*w*) on indicted markers in E2-mediated mammary tumorigenesis. Total RNA was isolated by Trizol method and quantified by Nanodrop. One-Step SYBR green qRT-PCR Kit (Quanta Biosciences, Gaithersburg, MD, USA) was used to perform cDNA synthesis and PCR amplification simultaneously using 100 ng of total RNA as per manufacturer’s instructions. Relative gene expression was normalized to 18s rRNA and assessed using the differences in normalized Ct (^ΔΔ^*C*t). Fold changes were calculated by 2^−ΔΔ*C*t^. Experiments were performed in triplicate for each data point. Data represent mean ± SD of fold change in mRNA expression relative to the untreated control of five animals. Symbols (– and +) denotes the absence and presence of E2, respectively. Alphabets (a and b) followed by asterisk shows statistical differences analyzed by student’s *t* test where * *p* < 0.05; *** *p* < 0.001. ND, not determined.

To better understand the mechanism by which the Apiaceae spices inhibit mammary tumorigenesis, expression of E2-associated genes was analyzed in mammary tissues at 12 weeks by qRT-PCR and western blot analysis following the spice intervention in the absence and presence of E2. To understand the metabolism of E2, levels of CYP1A1 and 1B1 were also measured. E2 treatment significantly upregulated CYP1A1 by almost five-fold whereas 1B1 was down-regulated by four-fold. All the spices favorably modulated the expression of 1A1 and 1B1, except celery which enhanced the expression level of 1A1 (Figure 5).

Similarly, expression of estrogen receptor α (ERα) was significantly increased by E2 treatment (over three-fold) and was down-regulated by all spice treatments. The effect was most pronounced with caraway (Figure 5). The E2-associated increase was also found in the regulation of cyclin D1. Anise and caraway offset the expression of cyclin D1, but the reductions were statistically insignificant.

### 2.6. Tumor Incidence, Volume, and Multiplicity

**Figure 6:**
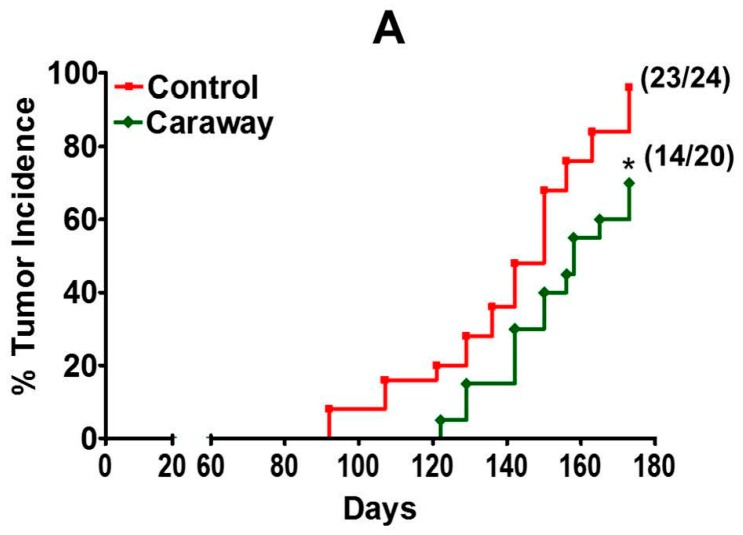

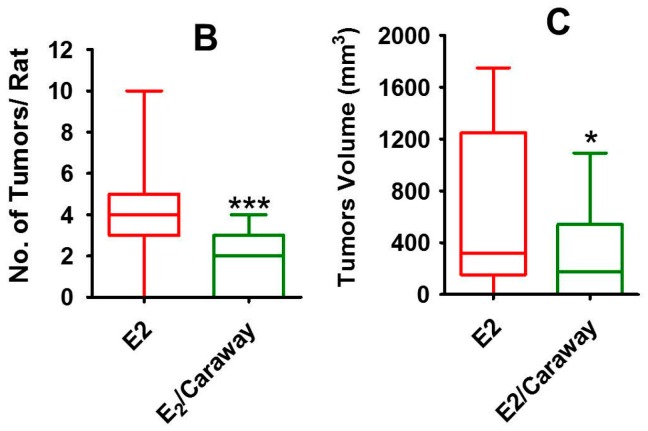
The effect of caraway supplemented diet (7.5%) on tumor indices. After challenging with E2 implants, female ACI rats were palpated starting 12 weeks. Incidence (**A**) were analyzed by nonparametric log-rank test. Tumor multiplicity (**B**) and tumor volume (**C**) were calculated at the time of euthanasia. Statistical analysis was done by two-tailed student’s *t*-test. * *p* < 0.05; *** *p* < 0.001.

Based on the biomarker study results, the caraway (7.5% *w*/*w*) treatment was continued to test antitumor activity. At the study termination (25 weeks), caraway intervention delayed tumor latency 29 days (Figure 6A). At the time of euthanasia, 23 of 24 rats (96%) had palpable mammary tumors in the E2-treated animals whereas only 70% of the rats receiving caraway diet intervention developed tumors. Furthermore, the caraway diet significantly inhibited tumor multiplicity by almost three-fold (*p* = 0.0001) with 1.75 tumors/rats compared with 4.2 tumors/rat in E2-treated control (Figure 6B). Similarly, the tumor volume was reduced over 50% by the caraway diet (313 ± 81 mm^3^) compared to the control group (661 ± 123 mm^3^) (Figure 6C).

### 2.7. Potential Toxicity of Caraway

To test the potential toxicity of caraway, animals treated with caraway supplemented diet (7.5%) for 25 weeks in the absence of E2 were analyzed. No gross toxicity was observed during the study and there were no adverse effects on the body weight gain, mobility, or diet consumption. Blood analysis (Table 2) indicated that the caraway diet did not affect liver and kidney functions as well as hematopoietic parameters compared to untreated controls. The differences observed in the Alkaline phosphatase, blood urea nitrogen (BUN)/creatinine and hematocrit (HCT) (Table 2 and Table 3) are due to the E2 treatment irrespective of control/caraway diet or were under the physiological limits.

## 3. Discussion

Epidemiological studies suggest that women with higher circulating levels of the female hormone, E2 are at higher risk of developing breast cancer. E2 and its hydroxylated metabolites resulting from CYP1B1 and CYP1A1 have been implicated in breast cancer. The ability to suppress breast-cancer growth by ovariectomy was demonstrated even in the 18th century in young women [34]. Furthermore, prevention and treatment of breast cancer is possible using selective ER modulator (SERM), tamoxifen and others [35]. However, risks such as increased incidence of endometrial cancer with tamoxifen treatment [36], development of drug-resistant tumors [37], as well as cross-reactivity and treatment failure [38] are known limitations. Thus, additional therapeutics are warranted that not only modulate ER but also inhibit E2 metabolism.

Phytochemicals derived from edible and medicinal plants are attractive for prevention and treatment of breast and other cancers because of their efficacy in preclinical models and favorable safety profiles [39]. Studies from our laboratory indicate extracts of the Apiaceae spices (anise, caraway, coriander, cumin, dill, and fennel) are protective against oxidative DNA adducts resulting from redox activity of E2 catechols, and they also inhibit cytochrome P450 activity associated with E2 metabolism [32].

Disease progression can be understood as the decline in health of the animals as indicated by body weight loss, altered diet intake, and morbidity score. The importance of organ systems, such as the liver, must also be taken into account when analyzing the mechanisms by which E2 induces mammary tumors. In this study, the spice interventions were well tolerated and no effect on body and organ weights was observed. E2 treatment increased the body weight compared to age-matched untreated control, consistent with our previous studies [40,41,42].

One of the major pathways proposed for E2-induced mammary tumorigenesis is through E2 activated Estrogen Receptor-mediated gene expression, which leads to proliferation of mammary cells. As shown by our laboratory and others, E2 induces cell proliferation by five- to six-fold compared with vehicle control at 12 weeks, as measured by immunostaining the mammary tissues for PCNA. All the test spices demonstrated significant antiproliferative activity against E2-induced mammary proliferation. The antiproliferative effects of spices and pure compounds isolated from various spices have been studied on multiple cancer cell types (as reviewed in [43]). E2 is proliferative primarily through its action on ERα, which is also required for normal development and differentiation of the mammary gland [35,44] The spice intervention reduced the E2-induced levels of ERα, suggesting that phytochemicals present in these spices are potent anti-estrogenic and potentially interact with the ERα pathway. Interestingly, dietary spice intervention does not completely block ER, but did return ER to control levels. Cyclin D1 is overexpressed in more than 50% of breast cancers, functioning as a rate-limiting factor for human breast cancer cell proliferation in vivo and in vitro [45,46]. E2-induced upregulation of cyclin D1 plays an important role in cell proliferation and cell cycle transition. Dietary intervention with all the test spices offset the E2-mediated increases in the cyclin D1 expression. Thus, spice intervention effectively reversed the proliferative effect of E2 treatment. Another major pathway of E2-induced tumorigenesis is the oxidative damage induced by the 2E2 and highly carcinogenic 4E2 hydroxyl metabolites of E2. We previously demonstrated that the hydroxyl metabolites of E2 induce extensive oxidative damage to the DNA by redox cycling in the presence of divalent cation [47]. Our additional studies also demonstrate that various extracts of spices modulated the estrogen metabolism pathway at different stages. Extracts of spices modulate the enzyme activity of CYP450s as well as scavenge the free radicals generated by the redox-cycling of the hydroxyl metabolites of E2 [32]. In the current study, we demonstrate similar results in short-term in vivo biomarker studies that suggest the phytochemicals from the spices are bioavailable and bioactive at target mammary tissue.

E2 and the resulting hydroxylated metabolites from cytochrome P450s CYP1A1 and CYP1B1 have been implicated in breast cancer. CYP1A1 forms both 2E2 and 4E2 [48] whereas human CYP1B1, which is highly expressed in E2 target tissues, catalyzes the metabolic activation of various procarcinogens and the 4-hydroxylation of E2 [49,50]. Further, human microsomes from mammary adenocarcinoma and fibroadenoma predominantly catalyze the 4-hydroxylation of E2 [51]. Thus, enhanced levels of 4E2 are suggested to play a mechanistic role in tumor development in hormone-sensitive organs. Dietary exposure to Apiaceae spices were able to offset modulation of cytochrome P450s associated with E2 treatment and thus potentially reduced the DNA damage caused by its genotoxic catechol-estrogen metabolites.

Mammary gland development is affected by three major hormones: estrogen, progesterone, and prolactin. Dietary exposure to Apiaceae spices did not affect endogenous levels of E2. However, spice interventions significantly reduced E2 levels in animals supplemented with E2, suggesting that these spices can alter circulating levels of E2. However, chemopreventive agents can also act without altering the circulating E2 levels [52] through their interaction with pituitary. Prolactin plays an important role in mammary gland development and lactation and has been implicated in the etiology of breast cancer [53]. In the present study, female ACI rats exhibited increased pituitary weight and circulating prolactin in response to E2 treatment. Since dietary exposure to Apiaceae spices reduced this level compared to E2 controls these spices may be effective against this axis of tumor promotion.

Collectively, dietary exposure of ACI rats to Apiaceae spices affected the development of mammary tumorigenesis by reversing E2-induced changes in cytochrome P450s (catechol pathway) and by affecting PCNA, E2 receptor, and cyclin D1 (cell proliferation pathway) in addition to the effect on circulatory E2 and prolactin. Thus, compounds present in the Apiaceae spices may significantly reverse the tumor-initiating effects of E2 by affecting the phase I metabolism of E2 and by reducing the DNA damage caused by its genotoxic catechol-estrogen metabolites. Future work should be focused on the identification of the therapeutic phytochemicals from these compounds.

Based on the early biomarkers of proliferation and modulation of cytochrome P450s, we chose caraway for a longer-term tumorigenesis study. One of the hallmarks of tumor prevention is increased tumor latency of in vivo models. Caraway not only increased tumor latency by 29 days, but also significantly reduced the mammary tumor incidence, volume, and multiplicity. Based on these results, it is clear the effect on tumors is also reflected in decreased proliferative indices. The favorable modulation of all of the three biomarkers related to proliferation we studied, PCNA, cyclin D1, and ERα suggests that dietary caraway can be an effective chemopreventive approach in high risk groups and cancer survivors. Even though we have not analytically analyzed caraway for therapeutic phytochemicals, others have shown that thymoquinones isolated from caraway have similar activities [54]. Studies are planned in our laboratory to identify additional potential therapeutic phytochemicals from caraway.

Toxic side-effects of various drugs in clinical trials hamper progress of many potential drugs. We report 7.5% *w*/*w* of caraway in the diet was well tolerated by the rats and pathological examinations of various tissue indicated no gross tissue toxicity. A lack of systemic toxicity based on blood chemistry is also a good indicator of dietary tolerance of caraway. Thus, we propose dietary caraway should be further explored as an effective approach for prevention of multiple cancers where E2 plays a significant etiological factor.

## 4. Material and Methods

### 4.1. Chemicals

Seeds of anise (*Pimpinella anisum*), caraway (*Carum carvi*), and celery (*Apium graveolens*) (Figure 1A) were purchased from Frontier Herbs (Danbury, CT, USA). E2 was purchased from Steraloids, Inc. (Newport, RI, USA). The silastic tube (2.0 id. × 3.2 od. mm) was purchased from Allied Biomedical, Inc. (Ventura, CA, USA) and medical-grade silicone adhesive was purchased from Factor II, Inc. (Lakeside, AZ, USA). Anti-Estrogen receptor α (ERα) and anti-progesterone receptor antibodies were purchased from Santa Cruz Biotechnology (SantaCruz, CA, USA), anti-cyclin D1 from Abcam (Cambridge, MA, USA), and anti-β-actin was purchased from Sigma-Aldrich (St. Louis, MO, USA). All other chemicals used were of analytical grade.

### 4.2. Diet

Purified AIN-93M diet was purchased in pellet form from Harlan–Teklad, Inc. (Madison, WI, USA). The dried spice seeds were finely powdered using a kitchen blender, sieved, and lyophilized to remove residual moisture. All spice powders were then vacuum packed and stored at −20 °C until use. Test diets containing anise, caraway, and celery (7.5% *w*/*w*) were custom made by Harlan-Teklad. The composition of the AIN-93M diet was modified for each diet and made isocaloric based on information provided on the United States Department of Agriculture (USDA) website (available online: http://www.nal.usda.gov/fnic). All diets were vacuum sealed in small packs and stored at 4 °C until used. The daily feed intake by animals was assessed by subtracting the final weight from the initial weight of diet on a weekly basis.

### 4.3. Animal Study

Five to six week-old female ACI rats were purchased from Envigo, formerly Harlan-Sprague-Dawley (Indianapolis, IN, USA). The animals received food and water ad libitum and all procedures followed were in accordance with an approved Institutional Animal Care and Use Committee (IACUC) protocol number 13008, dated 1 August 2013. E2 implants were prepared using silastic tubing containing 9.0 ± 0.2 mg E2 as previously described [42]. Two studies were conducted to determine the effects of Apiaceae spices against E2-induced mammary tumorigenesis.

In the first study, after a week of acclimatization, rats (*n* = 5–6) were randomized based on body weights into groups provided with either control AIN-93M diet or diet supplemented with anise, caraway, or celery seed powder (7.5% *w*/*w*) for 14 days prior to challenge with E2 implantation. One half of the animals in each group received E2 implants (1.2 cm; 9 ± 0.2 mg E2) subcutaneously as reported previously [40] and half were used as untreated controls. Animals (*n* = 6) from each group were euthanized by CO_2_ asphyxiation after 3 and 12 weeks of E2 treatment for biomarker study. Blood was removed and plasma and serum were separated to measure circulating E2, prolactin, and systemic toxicity. Mammary, and other tissues were collected, weighed, and snap-frozen in liquid nitrogen for future analyses. Frozen tissues were stored at −80 °C.

The second study was conducted to evaluate the effects of caraway (selected from the first study) on E2-induced mammary tumorigenesis. This study was patterned after the first study (Figure 1B). Female ACI rats (*n* = 20–25) were provided either control AIN-93M diet or diet supplemented with caraway (7.5% *w*/*w*). Starting from the 12th week, animals were palpated for mammary tumors. The tumor size was measured weekly by Vernier Caliper. Once tumor incidence in control animals reached 90%–95%, the study was terminated and all animals were euthanized by CO_2_ asphyxiation followed by bilateral thoracotomy. Tumor incidence and the number of tumors per rat were counted at the time of dissection. Tumor tissues and organs were collected and snap frozen at −80 °C.

### 4.4. Evaluation of Cell Proliferation by Immunohistochemistry

Antiproliferative staining was done as described previously [55]. Briefly, at the time of euthanasia, portions of normal mammary and tumor tissues were stored in 10%-buffered formalin. The samples were transferred to 70% ethanol the next day for histopathologic analyses. Formalin-fixed tissues were embedded in paraffin, and 4–5 µm sections were cut. Sections of the mammary tissues were stained with hematoxylin and eosin (H&E) or proliferating cell nuclear antigen (PCNA) using Zymed PCNA kit (Invitrogen Co., Carlsbad, CA, USA) following manufacturer’s guidelines as described previously [55]. Counterstained sections were blinded and evaluated under a bright-field microscope (Nikon Eclipse 80i, Melville, NY, USA) and deeply-stained cells were counted by two independent pathologists. Under each field, 100 cells were counted and 10 such fields were scored and average values presented. Images were acquired using Nikon NIS-Elements software (version 3.10 SP3, Melville, NY, USA).

### 4.5. Serum E2 Analysis

Serum samples were analyzed for circulating E2 levels by electrochemi-luminescent detection using the Roche E170 immunoassay analyzer at the University of Louisville Hospital’s Clinical Chemistry facility as previously described [40]. All samples were analyzed in triplicate.

### 4.6. Plasma Prolactin

Blood plasma samples were collected at euthanasia and circulatory prolactin levels were analyzed. Prolactin analysis was performed by enzyme immune-assay kit (Alpco Diagnostics, Windham, NH, USA) as per the manufacturer’s instructions.

### 4.7. Western Blot Analysis

Whole cell lysates (WCL) were prepared from mammary tissues using radioimmunoprecipitation assay (RIPA) buffer (Santa Cruz Biotechnology, Santa Cruz, CA, USA). The expression of proteins was analyzed by western-blot analysis as described [2,56]. Proteins were transfer to polyvinylidene difluoride (PVDF) membrane using semi-dry transfer apparatus, and the membrane was immunoblotted with anti-cyclin D1, -ERα, and -PCNA antibodies (appropriate secondary antibodies were used and detection was carried out using an enhanced chemiluminescence reagent (Thermo Scientific, Waltham, MA, USA)). Equal loading of the proteins was confirmed using β-actin (Sigma-Aldrich, St. Louis, MO, USA).

### 4.8. Real-Time PCR for Target Gene Expression

Primers for qRT-PCR were designed using Primer Express 3.0 software and synthesized by Integrated DNA Technologies, Inc. as previously described [57]. The sequences of the forward and reverse primers for each gene tested are: CYP1A1 forward, 5′-TGGAGACCTTCCGACATTCAT-3′; reverse, 5′-GGGATATA GAAGCCATTCAGACTTG-3′; CYP1B1 forward, 5′-AACCCAGAGGACTTT GATCCG-3′; reverse, 5′-CGTCGTTTGCCCACTGAAAA-3′; cyclinD1 forward, 5′-CCAGCCTTCTGACCTCTTTCC-3′; reverse, 5′-TCTTCGATTTGTTTTGCATCCA-3′, ERα forward 5′-GGCACATGAGTAACAAAGGCA-3′; reverse 5′-GGCATGAAGACGATGAGCAT-3′, A One-Step SYBR green qRT-PCR Kit (Quanta Biosciences, Gaithersburg, MD, USA) was used as per manufacturer’s instructions. PCR conditions were a hold at 50 °C for 10 min, 95 °C for 5 min, then 40 cycles at 95 °C for 10 s, and 60 °C for 30 s. Relative gene expression was assessed using the differences in normalized *C*_t_ (ΔΔ*C*_t_) method after normalization to 18 s rRNA. Fold changes were calculated by 2^−ΔΔ*C*t^. Experiments were performed in triplicate for each data point.

### 4.9. Toxicity Testing

Blood was collected separately at the time of euthanasia (tumor time point; 25 weeks) and toxicology profile was analyzed as described earlier [41].

### 4.10. Statistical Analyses

Student t-test was performed to examine the differences between mean of the treatment for body weight, organ weights, serum E2 levels, and tumor volume/multiplicity, etc. Tumor multiplicity was analyzed using the Negative-Binomial-regression model with logarithmic link. All analyses were done using Graph pad prism software (La Jolla, CA, USA). Tumor latency was determined by Kaplan-Meier survival estimates and plots. A *p*-value of <0.05 was considered significant for all of the assays.

## 5. Conclusions

In summary, spices from Apiaceae family are rich in phytochemicals capable of inhibiting mammary tumorigenesis. Data suggest that Apiaceae spices offset E2-mediated increase in mammary cell proliferation, ERα, cyclin D1, and CYP1A1/CYP1B1. The reduction of circulating E2 and prolactin levels by the spices is particularly noteworthy as these are some of the key factors in mammary tumorigenesis in this model [2,53]. These data strongly correlate with the observed anti-tumor activity against E2-induced mammary tumorigenesis. The wide use of these spices in cuisines and lack of toxicity demonstrated in this study further suggest the feasibility of the Apiaceae spices as potent cancer chemopreventive agents.

## Figures and Tables

**Table 1:** Animal body and organ weights after 3 and 12 weeks of intervention.

Group	Weights (g) at 3 Weeks				Weights (g) at 12 Weeks			
	Body wt.	Liver	Mammary	Pituitary ^a^	Body wt.	Liver	Mammary	Pituitary ^a^
Control diet	153.2 ± 9.3	4.5 ± 0.4	3.9 ± 1.1	8.5 ± 1.2	169 ± 9	4.4 ± 0.4	4.0 ± 0.6	8.7 ± 0.3
Anise	159.1 ± 4.0	5.1 ± 0.4	4.6 ± 1.0	7.7 ± 1.9	172 ± 3	4.9 ± 0.2	3.3 ± 0.2	9.4 ± 1.0
Caraway	157.5 ± 2.4	4.6 ± 0.1	4.0 ± 0.4	9.2 ± 0.5	166 ± 3	4.6 ± 0.1	2.6 ± 0.3	9.2 ± 0.9
Celery	146.3 ± 4.7	5.4 ± 0.3	3.2 ± 0.5	7.7 ± 1.0	171 ± 6	5.4 ± 0.3	4.4 ± 1.2	8.6 ± 1.4
E2 + Control diet	156.0 ± 16	7.0 ± 0.4 ***	4.9 ± 1.2 **	17.9 ± 2.6 ***	184 ± 13 *	6.3 ± 0.2 **	6.5 ± 0.9 ***	32.5 ± 2.4 ***
E2 + Anise	159.5 ± 6.2	6.8 ± 0.3	5.4 ± 0.9	19.1 ± 3.9	177 ± 9	7.1 ± 0.7	5.5 ± 0.4	26.6 ± 5.5 ^#^
E2 + Caraway	160.3 ± 3.5	7.2 ± 0.5	4.3 ± 0.6 ^#^	20.6 ± 5.0	184 ± 3	7.7 ± 0.3	6.7 ± 0.2	21.2 ± 5.7 ^###^
E2 + Celery	158.0 ± 6.0	7.8 ± 0.6	4.9 ± 0.5	14.4 ± 3.5 ^#^	187 ± 14	9.0 ± 0.8	6.3 ± 0.4	21.8 ± 5.2 ^###^

^a^ Pituitary weights are represented in mg. Asterisks show significant difference at * *p* < 0.05; ** *p* < 0.01; *** *p* < 0.001 versus the control animals whereas ^#^ denotes comparison between E2/spice to E2 treated control animals at ^#^ *p* < 0.05; ^###^ *p* < 0.001. E2, 17β-estradiol.

**Table 2:** The effects of E2 exposure and caraway supplemented diet (7.5%) on biochemical profiles (systemic toxicity) at the tumor time point in ACI rats.

Biochemical Profile	Control	Caraway	E2/Control	E2/Caraway
Liver profile				
Aspartate aminotransferase	154 ± 67	203.3 ± 79.3	207.5 ± 88.8	154.7 ± 34.7
Alanine aminotransferase	51 ± 9.1	47.7 ± 18.0	37.3 ± 6.6 *	45.7 ± 5.3
Alk Phosphatase	51 ± 7.3	49.7 ± 12.6	27.3 ± 6.1 **	32.3 ± 2.2 **
Gamma-glutamyl transferase	1 ± 0.5	1.3 ± 0.5	2.3 ± 0.8	1.5 ± 1
Amylase	654 ± 58	584 ± 18	561 ± 47	670 ± 50
Creatine phosphokinase	798 ± 295	1017 ± 330	1010 ± 549	634.7 ± 127
Kidney profile				
Blood Urea nitrogen (BUN)	28.5 ± 2.4	18.7 ± 1.5	25.7 ± 2.3	22.3 ± 3.6
BUN/Creatinine Ratio	61.5 ± 6	40.7 ± 8.3 *	55.7 ± 8.8	40.5 ± 6.1 *
Phosphorus	15.2 ± 0.9	18.1 ± 2.1	12.5 ± 1.4	12.9 ± 0.6
Calcium	10.9 ± 0.9	10.5 ± 1.1	11.5 ± 0.3	11.9 ± 0.1
Total Protein	8.1 ± 0.4	7.0 ± 0.4	8.0 ± 0.3	8.1 ± 0.3
Albumin	4.9 ± 0.3	4.1 ± 0.3	4.7 ± 0.3	4.5 ± 0.2
Globulin	3.3 ± 0.1	2.9 ± 0.1	3.3 ± 0.4	3.7 ± 0.05
Albumin/Globulin Ratio	1.5 ± 0.1	1.43 ± 0.2	1.4 ± 0.3	1.2 ± 0.08
Glucose	154.7 ± 40.5	128.7 ± 42.1	107.8 ± 7.1	109.7 ± 27.5
Cholesterol	158.3 ± 39.7	101.7 ± 1.53 *	120.8 ± 8.7	125.5 ± 9.3
Triglyceride	203.7 ± 28.1	114.7 ± 36.5 *	107.0 ± 44.7	171.2 ± 91.6

Female ACI rats (five to six weeks old) were provided control diet (AIN 93M) and water ad libitum or treated with caraway (7.5% *w*/*w*) diet in the absence and presence of E2 by oral gavage for 25 weeks, once daily. At euthanasia, blood was collected and analyzed using an automated AU640^®^ Chemistry Analyzer (Beckman Coulter, Inc., Brea, CA, USA) by Antech diagnostics. Data represent the average of four animals. Statistical analysis was performed by student *t*-test. * *p* < 0.05; ** *p* < 0.01.

**Table 3:** The effects of E2 exposure and caraway supplemented diet (7.5%) on the hematological parameters (systemic toxicity) at the tumor time point in ACI rats.

Hematological Profile	Control	Caraway	E2/Control	E2/Caraway
White blood cells	5.1 ± 1.1	4.8 ± 1.6	4.4 ± 1.5	4.5 ± 1.2
Hemoglobin	13.3 ± 0.6	12.2 ± 0.2	10.5 ± 0.9	11.5 ± 0.8
Hematocrit	46.7 ± 1.2	41.7 ± 1.5	34.8 ± 4.1 **	38.5 ± 2.4 *
Mean corpuscular volume	56 ± 1.0	54.3 ± 1.2	52.8 ± 2.5	54.5 ± 2.1
Mean corpuscular hemoglobin conc.	28.7 ± 0.6	29.7 ± 0.6	30.2 ± 1.6	29.8 ± 1.0
Platelet Count	939 ± 61	993.7 ± 61.7	745.3 ± 208	845 ± 24.7
Neutrophils	17.8 ± 7.5	14.7 ± 2.1	28 ± 11.5	17.0 ± 7.4
Lymphocytes	76.8 ± 7.8	81 ± 1.7	64.8 ± 12.6	75.3 ± 5

Female ACI rats (five to six weeks old) were provided control diet (AIN 93M) and water ad libitum or treated with caraway (7.5% *w*/*w*) diet in the absence and presence of E2 by oral gavage for 25 weeks, once daily. At euthanasia, blood was collected and analyzed using an automated AU640^®^ Chemistry Analyzer (Beckman Coulter, Inc., Brea, CA, USA) by Antech diagnostics. Data represent average of four animals. Statistical analysis was performed by student’s *t*-test and no significant change was observed control group. * *p* < 0.05; ** *p* < 0.01.

## Abbreviations

SERM	Selective E2-receptor modulators
PCNA	Proliferating cell nuclear antigen
E2	17β-estradiol
ACI	August-Copenhagen Irish rats
ERα	Estrogen receptor α
BC	Breast cancer
HRT	Hormone replacement therapy
4E2	4-Hydroxyestrone
4E1	4-Hydroxyestrone

## References

[1] American Cancer Society (2017). Cancer Facts & Figures 2016.

[2] Chen W.Y. (2008). Exogenous and endogenous hormones and breast cancer. Best Pract. Res. Clin. Endocrinol. Metab..

[3] Cummings S.R., Duong T., Kenyon E., Cauley J.A., Whitehead M., Krueger K.A. (2002). Serum estradiol level and risk of breast cancer during treatment with raloxifene. JAMA.

[4] Boyd N.F., Guo H., Martin L.J., Sun L., Stone J., Fishell E., Jong R.A., Hislop G., Chiarelli A., Minkin S. (2007). Mammographic density and the risk and detection of breast cancer. N. Engl. J. Med..

[5] Krishnan K., Baglietto L., Apicella C., Stone J., Southey M.C., English D.R., Giles G.G., Hopper J.L. (2016). Mammographic density and risk of breast cancer by mode of detection and tumor size: A case-control study. Breast Cancer Res..

[6] Yaghjyan L., Colditz G.A., Collins L.C., Schnitt S.J., Rosner B., Vachon C., Tamimi R.M. (2011). Mammographic breast density and subsequent risk of breast cancer in postmenopausal women according to tumor characteristics. J. Natl. Cancer Inst..

[7] O’Connor M., Rhodes D., Hruska C. (2009). Molecular breast imaging. Expert Rev. Anticancer Ther..

[8] Stefansson O.A., Jonasson J.G., Johannsson O.T., Olafsdottir K., Steinarsdottir M., Valgeirsdottir S., Eyfjord J.E. (2009). Genomic profiling of breast tumours in relation to brca abnormalities and phenotypes. Breast Cancer Res..

[9] Hartmann L.C., Schaid D.J., Woods J.E., Crotty T.P., Myers J.L., Arnold P.G., Petty P.M., Sellers T.A., Johnson J.L., McDonnell S.K. (1999). Efficacy of bilateral prophylactic mastectomy in women with a family history of breast cancer. N. Engl. J. Med..

[10] Rebbeck T.R., Friebel T., Lynch H.T., Neuhausen S.L., van’t Veer L., Garber J.E., Evans G.R., Narod S.A., Isaacs C., Matloff E. (2004). Bilateral prophylactic mastectomy reduces breast cancer risk in *BRCA1* and *BRCA2* mutation carriers: The PROSE Study Group. J. Clin. Oncol..

[11] Lostumbo L., Carbine N.E., Wallace J. (2010). Prophylactic mastectomy for the prevention of breast cancer. Cochrane Database Syst. Rev..

[12] Dale L.C., Gotay C.C. (2012). The relationship between complementary and alternative medicine use and breast cancer early detection: A critical review. Evid. Based Complement. Altern. Med..

[13] Wanchai A., Armer J.M., Stewart B.R. (2010). Complementary and alternative medicine use among women with breast cancer: A systematic review. Clin. J. Oncol. Nurs..

[14] Vogel V.G. (2011). Update on raloxifene: Role in reducing the risk of invasive breast cancer in postmenopausal women. Breast Cancer.

[15] Nichols H.B., DeRoo L.A., Scharf D.R., Sandler D.P. (2015). Risk-benefit profiles of women using tamoxifen for chemoprevention. J. Natl. Cancer Inst..

[16] Chen J.Y., Kuo S.J., Liaw Y.P., Avital I., Stojadinovic A., Man Y.G., Mannion C., Wang J., Chou M.C., Tsai H.D. (2014). Endometrial cancer incidence in breast cancer patients correlating with age and duration of tamoxifen use: A population based study. J. Cancer.

[17] Oseni T., Patel R., Pyle J., Jordan V.C. (2008). Selective estrogen receptor modulators and phytoestrogens. Planta Med..

[18] Wang J., Jiang Y.F. (2012). Natural compounds as anticancer agents: Experimental evidence. World J. Exp. Med..

[19] Low Dog T. (2006). A reason to season: The therapeutic benefits of spices and culinary herbs. Explore.

[20] Kaefer C.M., Milner J.A. (2008). The role of herbs and spices in cancer prevention. J. Nutr. Biochem..

[21] Boskabady M.H., Alitaneh S., Alavinezhad A. (2014). *Carum copticum* L.: A herbal medicine with various pharmacological effects. BioMed Res. Int..

[22] Tapsell L.C., Hemphill I., Cobiac L., Patch C.S., Sullivan D.R., Fenech M., Roodenrys S., Keogh J.B., Clifton P.M., Williams P.G. (2006). Health benefits of herbs and spices: The past, the present, the future. Med. J. Aust..

[23] Ji H.F., Li X.J., Zhang H.Y. (2009). Natural products and drug discovery. Can thousands of years of ancient medical knowledge lead us to new and powerful drug combinations in the fight against cancer and dementia?. EMBO Rep..

[24] Cragg G.M., Newman D.J. (2013). Natural products: A continuing source of novel drug leads. Biochim. Biophys. Acta.

[25] Davis D.L., Telang N.T., Osborne M.P., Bradlow H.L. (1997). Medical hypothesis: Bifunctional genetic-hormonal pathways to breast cancer. Environ. Health Perspect..

[26] Foster J.S., Henley D.C., Ahamed S., Wimalasena J. (2001). Estrogens and cell-cycle regulation in breast cancer. Trends Endocrinol. Metab..

[27] Lombardi M., Castoria G., Migliaccio A., Barone M.V., Di Stasio R., Ciociola A., Bottero D., Yamaguchi H., Appella E., Auricchio F. (2008). Hormone-dependent nuclear export of estradiol receptor and DNA synthesis in breast cancer cells. J. Cell Biol..

[28] Weroha S.J., Li S.A., Tawfik O., Li J.J. (2006). Overexpression of cyclins D1 and D3 during estrogen-induced breast oncogenesis in female ACI rats. Carcinogenesis.

[29] Forman M.R., Hursting S.D., Umar A., Barrett J.C. (2004). Nutrition and cancer prevention: A multidisciplinary perspective on human trials. Annu. Rev. Nutr..

[30] Willett W.C. (1995). Diet, nutrition, and avoidable cancer. Environ. Health Perspect..

[31] Martin K.R. (2006). Targeting apoptosis with dietary bioactive agents. Exp. Biol. Med..

[32] Jeyabalan J., Aqil F., Soper L., Schultz D.J., Gupta R.C. (2015). Potent chemopreventive/antioxidant activity detected in common spices of the apiaceae family. Nutr. Cancer.

[33] Dennison K.L., Samanas N.B., Harenda Q.E., Hickman M.P., Seiler N.L., Ding L., Shull J.D. (2015). Development and characterization of a novel rat model of estrogen-induced mammary cancer. Endocr. Relat. Cancer.

[34] Beatson G.T. (1983). On the treatment of inoperable cases of carcinoma of the mamma: Suggestions for a new method of treatment, with illustrative cases. Cancer J. Clin..

[35] Sims A.H., Howell A., Howell S.J., Clarke R.B. (2007). Origins of breast cancer subtypes and therapeutic implications. Nat. Clin. Pract. Oncol..

[36] Cano A., Hermenegildo C. (2000). The endometrial effects of serms. Hum. Reprod. Updat..

[37] Milano A., Dal Lago L., Sotiriou C., Piccart M., Cardoso F. (2006). What clinicians need to know about antioestrogen resistance in breast cancer therapy. Eur. J. Cancer.

[38] Lewis-Wambi J.S., Jordan V.C. (2005). Treatment of postmenopausal breast cancer with selective estrogen receptor modulators (SERMs). Breast Dis..

[39] Vyas A.R., Singh S.V. (2014). Molecular targets and mechanisms of cancer prevention and treatment by withaferin a, a naturally occurring steroidal lactone. AAPS J..

[40] Aqil F., Jeyabalan J., Munagala R., Singh I.P., Gupta R.C. (2016). Prevention of hormonal breast cancer by dietary jamun. Mol. Nutr. Food Res..

[41] Jeyabalan J., Aqil F., Munagala R., Annamalai L., Vadhanam M.V., Gupta R.C. (2014). Chemopreventive and therapeutic activity of dietary blueberry against estrogen-mediated breast cancer. J. Agric. Food Chem..

[42] Ravoori S., Vadhanam M.V., Sahoo S., Srinivasan C., Gupta R.C. (2007). Mammary tumor induction in Aci rats exposed to low levels of 17β-estradiol. Int. J. Oncol..

[43] Zheng J., Zhou Y., Li Y., Xu D.P., Li S., Li H.B. (2016). Spices for prevention and treatment of cancers. Nutrients.

[44] Dickson R.B., Stancel G.M. (2000). Estrogen receptor-mediated processes in normal and cancer cells. J. Natl. Cancer Inst. Monogr..

[45] Fu M., Wang C., Li Z., Sakamaki T., Pestell R.G. (2004). Minireview: Cyclin D1: Normal and abnormal functions. Endocrinology.

[46] Lee R.J., Albanese C., Fu M., D’Amico M., Lin B., Watanabe G., Haines G.K., Siegel P.M., Hung M.C., Yarden Y. (2000). Cyclin D1 is required for transformation by activated neu and is induced through an E2F-dependent signaling pathway. Mol. Cell. Biol..

[47] Spencer W.A., Vadhanam M.V., Jeyabalan J., Gupta R.C. (2012). Oxidative DNA damage following microsome/Cu(II)-mediated activation of the estrogens, 17β-estradiol, equilenin, and equilin: Role of reactive oxygen species. Chem. Res. Toxicol..

[48] Kisselev P., Schunck W.H., Roots I., Schwarz D. (2005). Association of CYP1A1 polymorphisms with differential metabolic activation of 17β-estradiol and estrone. Cancer Res..

[49] Tsuchiya Y., Nakajima M., Takagi S., Taniya T., Yokoi T. (2006). Microrna regulates the expression of human cytochrome P450 1B1. Cancer Res..

[50] Modugno F., Knoll C., Kanbour-Shakir A., Romkes M. (2003). A potential role for the estrogen-metabolizing cytochrome P450 enzymes in human breast carcinogenesis. Breast Cancer Res. Treat..

[51] Liehr J.G., Ricci M.J. (1996). 4-hydroxylation of estrogens as marker of human mammary tumors. Proc. Natl. Acad. Sci. USA.

[52] Li S.A., Weroha S.J., Tawfik O., Li J.J. (2002). Prevention of solely estrogen-induced mammary tumors in female ACI rats by tamoxifen: Evidence for estrogen receptor mediation. J. Endocrinol..

[53] Clevenger C.V., Furth P.A., Hankinson S.E., Schuler L.A. (2003). The role of prolactin in mammary carcinoma. Endocr. Rev..

[54] Sutton K.M., Greenshields A.L., Hoskin D.W. (2014). Thymoquinone, a bioactive component of black caraway seeds, causes G1 phase cell cycle arrest and apoptosis in triple-negative breast cancer cells with mutant p53. Nutr. Cancer.

[55] Ravoori S., Vadhanam M.V., Aqil F., Gupta R.C. (2012). Inhibition of estrogen-mediated mammary tumorigenesis by blueberry and black raspberry. J. Agric. Food Chem..

[56] Munagala R., Kausar H., Munjal C., Gupta R.C. (2011). Withaferin a induces p53-dependent apoptosis by repression of hpv oncogenes and upregulation of tumor suppressor proteins in human cervical cancer cells. Carcinogenesis.

[57] Munagala R., Aqil F., Vadhanam M.V., Gupta R.C. (2013). Microrna “signature” during estrogen-mediated mammary carcinogenesis and its reversal by ellagic acid intervention. Cancer Lett..

